# Editorial: Workplace health promotion

**DOI:** 10.3389/fpubh.2022.1090333

**Published:** 2022-12-06

**Authors:** Leah Okenwa Emegwa, Danijela Gasevic

**Affiliations:** ^1^Department of Health Sciences, Swedish Red Cross University, Stockholm, Sweden; ^2^School of Public Health and Preventive Medicine, Monash University, Melbourne, VIC, Australia

**Keywords:** wellbeing, wellness packages, uptake, managers, occupational health and safety

Due to the amount of time spent at the workplace and the impact of work on health and wellbeing over an individual's life course, a strategic position of the workplace as an important arena for population health promotion cannot be over emphasized (1, 2). Several risk factors within the physical and psychosocial working environment, as well as inadequate organizational support, result in work-related health problems, and have psychosocial and economic implications for the individual, the family, the organization and the society (3, 4).

There are numerous international policy documents regarding occupational health promotion, for example, the Luxembourg declaration for Workplace Health Promotion (WHP), which recommends that WHP should be strategic (5). A strategic approach implies that WHP should be conducted in a systematic and continuous process of needs analysis, priority setting, planning, implementation, and evaluation. In recognition of the importance of worker health and a healthy working life, but also in line with numerous occupational health goals, it is important to understand issues connected to population health from a workplace perspective. This Research Topic aims to highlight among others, barriers, enabling factors, best practices, emerging problems, and other issues important for WHP.

In this Research Topic, about 95 researchers from across the globe contributed to a total of 15 articles that examined WHP from diverse perspectives. Article summaries are presented below.

To develop and validate a Work Health Culture Scale (WHCS) more suitable for Taiwan's health culture assessment, Chang et al. used a three-stage method: (1) reviewing literature and defining domains (2) item generation, and (3) validation test. The newly developed instrument was found to have appropriate reliability and validity. The authors conclude by recommending further research on validity and reliability of WHCS in diverse sectors as well as the relationship between WHCS and other WHP indicators.

Tian et al. explored a cross-sectional association between occupational stress and fatigue, while also examining the mediating role of psychological capital (PsyCap) among Chinese physicians. They observed a high prevalence of fatigue among Chinese physicians, and that two important components of PsyCap, self-efficacy, and resilience, play more important roles in the mediating effect. The study suggests that intervention strategies and measures to relieve fatigue should be focused on physicians' positive PsyCap improvement.

In the study by Sigblad et al. a total of 19 managers were interviewed to understand their perceptions of employees' WHP uptake as well as challenges encountered by managers in the execution of their WHP-related tasks. The results of the study indicate that factors at the individual level, nature of the WHP offered, and organizational level factors played a key role in WHP uptake. Furthermore, challenges encountered by managers in executing WHP were mostly at the organizational level. The authors conclude that addressing these modifiable factors may improve WHP uptake among employees.

Yang et al. investigated the mediating effect of psychological capital (PsyCap) on the association between perceived organizational support and work engagement among medical doctors in China's Liaoning Province. In this cross-sectional study, self-administered questionnaires were distributed to 1,009 medical doctors. Findings suggest that the participants had a low level of work engagement, but that perceived organizational support could indirectly improve vigor, dedication, and absorption, partially through mediator PsyCap.

Mainsbridge et al. conducted a randomized-controlled pilot study with repeated measures of self-reported job stress and mood states in which 43 police officers were exposed to movement microbreaks during work hours. Preliminary findings suggest that interrupting sedentary work with movement microbreaks may have beneficial effects on employees' mental health. The authors discuss the implications and future research of movement microbreaks for mitigating work-related stress among police and by extension, first responders.

Deady et al. explored the utility and evaluated the acceptability, feasibility, and preliminary efficacy of a modified version of the HeadGear Apprentice app designed to reduce depressive symptoms in an apprentice. Findings suggest that the app was an acceptable and well-received intervention when adapted to young apprentices, however, addressing issues related to improving engagement and adherence to the program would improve effectiveness.

The paper by Hazelzet et al. describes the protocol for a planned evaluation study regarding effectiveness and the implementation process of the intervention “Healthy Human Resources” (HHR) on the sustainable employability of low-educated employees. A protocol consists of intended methods for effect evaluation (including a budget impact analysis), and process evaluation. The authors hypothesize that by improving dialogue HHR will strengthen the sustainable employability of low-educated employees and that if proven effective for tackling the socioeconomic health gap, HHR can be recommended on a wider scale.

COVID-19 brought many challenges to health care systems, as many healthcare workers became infected due to lack of adequate protection. Liu et al. shared their protocol for ensuring the safety of healthcare workers, which successfully controlled COVID-19 infection in the orthopedic department.

Kernan et al. evaluated a company-sponsored WHP program in a sample of long-term care facilities (nursing homes). Data were collected *via* standardized, self-administered questionnaire completed by a total of 1,589 workers in five job categories from 18 facilities within a single company. Findings show that the average levels of psychological demands and social support at work were relatively high. Compared to centers with no programs, supervisor support was higher in centers with well-developed WHP programs. Workers in centers with well-developed programs had slightly lower average body mass index and slightly lower prevalence of non-smoking as well as regular aerobic exercise. The low-intensity, low-resourced workplace health promotion program used in this study benefited a few individuals but had only modest influence on average levels of the measured health indicators.

Eriksson and Dellve conducted a mixed-methods study to identify the outcomes of a Swedish system-based WPH education program for managers and investigate impact of the program on health-oriented leadership, improvement work, and employee wellbeing. They reported that health-oriented leadership, improvement work, work satisfaction, and vitality increased at workplaces that worked actively to implement WHP following the program. These were also associated with improved job satisfaction. Furthermore, work environment issues, developmental leadership, and social learning climate may be important process indicators to consider when developing comprehensive WHP interventions.

Murray et al. explored the effect of a physical exercise training intervention on neck and shoulder muscle function [i.e., maximal voluntary contraction (MVC) and rate of torque development (RTD)] among military helicopter pilots and crew-members who were randomized to either an exercise-training-group (ETG; *n* = 35) or a reference-group (REF; *n* = 34). While REF received no training, the ETG received 20 weeks of self-administered exercise training specifically tailored to target the neck and shoulder muscles. Findings suggest that physical exercise training improved MVC and RTD in the upper neck extensors. Adherence to training regularly was poor, so future studies should focus on the practical implementation of self-administered exercise training to improve adherence.

In their mixed-methods study, Skagert and Dellve critically analyzed and identified interacting mechanisms and obstacles behind failures of organizational WHP projects from system perspectives. Obstacles identified included governance by logics of distancing and detaching, no binding regulation of WHP, separated responsibility of results, narrow focus on delegated responsibilities, store-fronting a strategic model, keeping poor organizational preconditions, and support for developments and isolating WHP from other organizational developments. The following should be considered when developing WHP programs: (1) the uncertainty a distributed empowerment to all system levels may create; (2) the distributed impact to define the target and allow broader areas to be included in WHP; and (3) the integration into other development processes and not reducing the organizational WHP to the form of a project.

Ma et al. presented the features, causes, and outcomes of serious workplace violence (WPV) against healthcare providers in China. The prevalence of serious WPV among healthcare providers is high, with doctors being the victims in most instances (81%). Death, severe injury and hospitalization were the major outcomes of serious WPV, which may arise from poor patient–doctor relationships, overly stressed health providers in highly demanding hospitals, poorly educated/informed patients, insufficient legal protection, and poor communication. Measures and policies are needed to prevent serious workplace violence and ensure safety of healthcare providers in China.

A mixed methods design was employed in the study by Nelson et al. to assess feasibility, acceptability, and preliminary efficacy of incorporating a whole-person care model of health coaching into an employee wellness program (i.e., weight loss, smoking cessation) that is made available by an employer-sponsored health plan. Thirty-nine employees and covered spouses from Loma Linda University Health were recruited into a 12-week whole person care intervention (a combination of health coaching and health education) and examined for outcomes, such as participants' experience and biometric data. For the qualitative study, key informant interviews were obtained from three health coaches and six intervention participants recruited *via* random sampling. Findings identify positive behavior change effects of an employee health intervention based on a whole person care model of health coaching with integrated health education, and identify the need for methods to maintain behavior change (i.e., mHealth, peer-support) post-intervention.

Doty et al. used pre-intervention, post- intervention design to explore changes in mental health utilization among Kent State University (KSU) employees before and after Right Direction (RD), a component of a universal employee wellness program implemented at KSU in 2014. Compared to the pre-intervention period, increased awareness of available resources resulted in an increased number of employees seeking assistance and engaging in care in the post-intervention period. Findings suggest that the effects of RD may be realized over the long-term with follow-up enhancements such as workshops/informational sessions on mindfulness, stress management, resiliency training, and self-acceptance.

## Author contributions

All authors listed have made a substantial, direct, and intellectual contribution to the work and approved it for publication.
