# Corrigendum: Editorial: Workplace health promotion

**DOI:** 10.3389/fpubh.2023.1152675

**Published:** 2023-02-22

**Authors:** Leah Okenwa Emegwa, Danijela Gasevic

**Affiliations:** ^1^Department of Health Sciences, Swedish Red Cross University, Stockholm, Sweden; ^2^School of Public Health and Preventive Medicine, Monash University, Melbourne, VIC, Australia

**Keywords:** wellbeing, wellness packages, uptake, managers, occupational health and safety

In the published article, there was an error in the author list, and the order of author list. The author names were incorrectly presented as: Danijela Gasevic 1, Leah Okenwa Emegwa 2.

The correct author list should be: Leah Okenwa Emegwa^1^^*^ and Danijela Gasevic^**2**^

^1^ Department of Health Sciences, Swedish Red Cross University, Stockholm, Sweden

^2^ School of Public Health and Preventive Medicine, Monash University, Melbourne, VIC, Australia

The authors apologize for this error and state that this does not change the scientific conclusions of the article in any way. The original article has been updated.

